# Positron Emission Tomography With Fluorodeoxyglucose Incidental Detection of Colon Cancer in a Patient’s Follow-Up for Nasopharyngeal Carcinoma During the COVID-19 Pandemic: A Case Report

**DOI:** 10.7759/cureus.9939

**Published:** 2020-08-22

**Authors:** Mohammed Basendowah, Shahad Alshaynawi, Turki A Madani, Mutaz H Alabdulqader, Muatasaim Hakami

**Affiliations:** 1 Surgery, King Abdulaziz University Hospital, Jeddah, SAU; 2 Family Medicine, King Abdulaziz Medical City, National Guard Hospital - Health Affairs, Jeddah, SAU

**Keywords:** colorectal cancer, pet/ct, nasopharyngeal carcinoma

## Abstract

Colorectal cancer (CRC) is a type of widespread, deadly malignancy that took thousands of lives around the globe. In the last two decades, CRC represented the most common cancer among men and ranked third among women in Saudi Arabia. Positron emission tomography with fluorodeoxyglucose (FDG-PET), can incidentally detect malignancy, as in our case, FDG-PET disclosed high abnormal FDG far away from the first primary malignancy. The current case is of a 65-year-old female who was following up on her nasopharyngeal carcinoma (first primary). During her last management, FDG-PET was requested to find any FDG uptake in the nasopharyngeal region; stunning FDG uptake was incidentally found at the ascending colon diagnosed as early-stage (pT2N0) colon cancer. Colonoscopy was done and India Ink was injected to facilitate localizing the mass during the laparoscopic removal of the tumor, which was delayed due to the pandemic of COVID-19. This took place in March 2020 at King Abdul-Aziz University Hospital in Jeddah.

## Introduction

In Saudi Arabia, colorectal cancer (CRC) represents the most common cancer among men and the third most common among women [1]. Up to 3% to 5% of colorectal cancer is related to personal or family history [2]. Positron emission tomography with fluorodeoxyglucose (FDG-PET) can be applied to detect, diagnose, and follow up malignancies [3]. It can disclose incidental uptake across different organs, including the colon (as in our case), rectum, lung, etc. [4]. Multiple primary cancer (MPC) is rare. However, as diagnostic and therapeutic methods progress, the number of MPCs grows annually [5]. Here, we present a case of an unlucky lady who fought nasopharyngeal carcinoma and colon cancer during the COVID-19 pandemic.

## Case presentation

**Figure 1:**
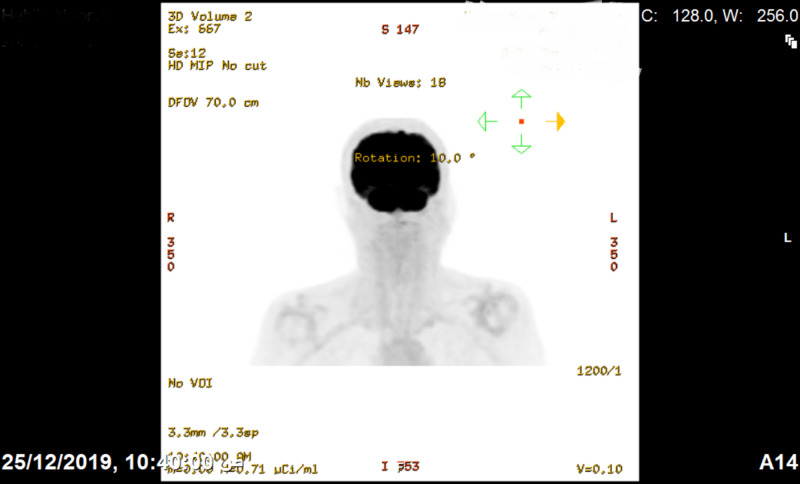
FDG-PET scan revealed no dominant FDG uptake in the nasopharyngeal region. FDG-PET: positron emission tomography with fluorodeoxyglucose

A 65-year-old female patient, known case of hypothyroidism, on thyroxin 75 mcg, dyslipidemia on Lipitor 20 mg, and hypertension on candesartan 16 mg. Unmarried and non-smoker with no family history of cancers, nor a past history of it. In May 2019, she was diagnosed with undifferentiated nasopharyngeal squamous cell carcinoma staged as cT2N0M0. After the head and neck tumor board discussion, the patient started her management with the first cycle of chemotherapy on May 19, 2019. The patient received three chemo cycles of ocetaxil + cisplatin followed by five cycles of carboplatin, which has ended curatively in October 2019. In the follow-up, magnetic resonance imaging (MRI) done on December 8, 2019, was negative for any residual or recurrence. For further evaluation, the FDG/PET scan revealed no dominant FDG uptake in the nasopharyngeal region (Figure 1).

**Figure 2:**
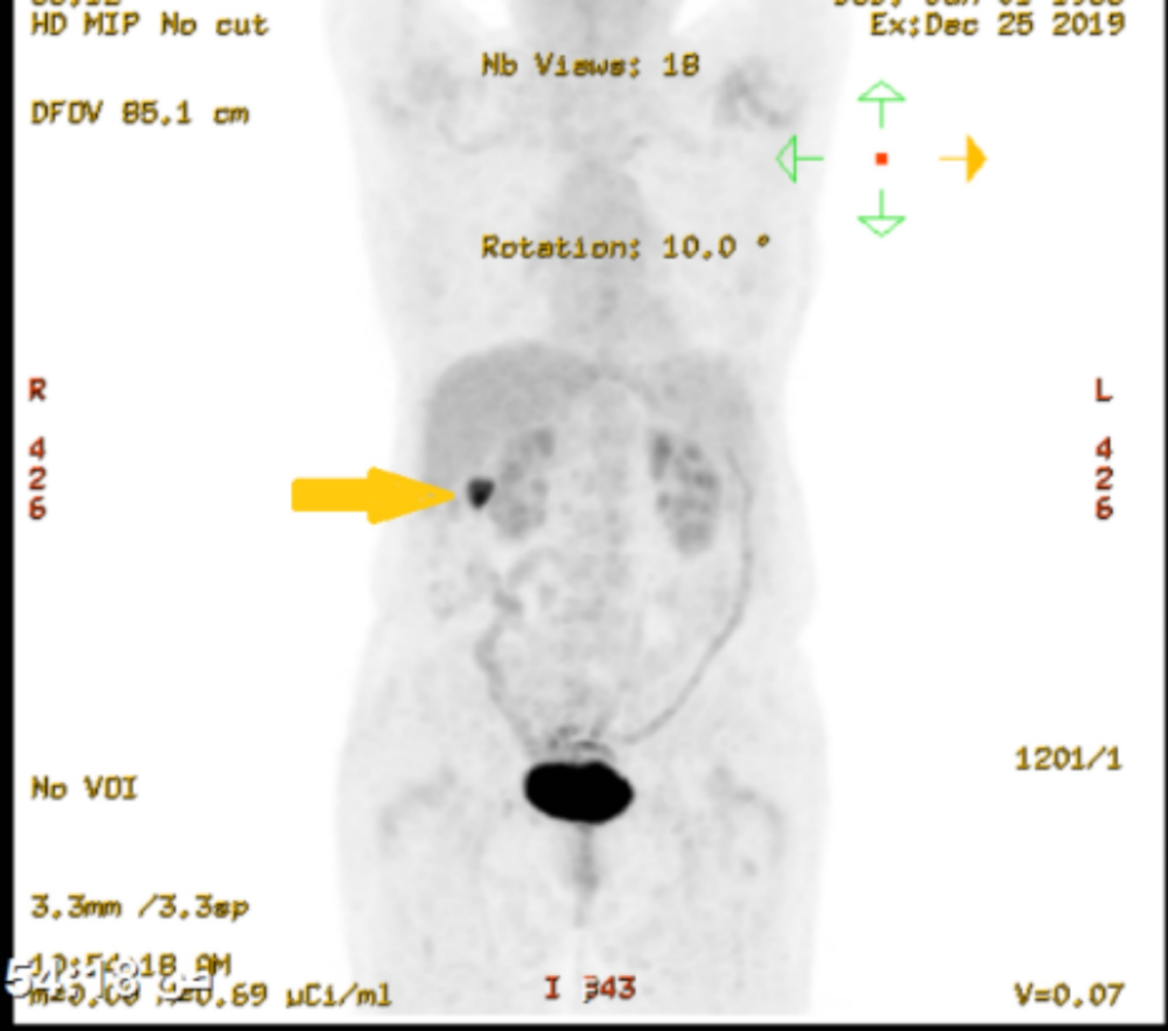
FDG-PET scan incidentally showing abnormal reuptake in the ascending colon FDG-PET: positron emission tomography with fluorodeoxyglucose

However, there was a focal intense FDG uptake at the right ascending colon (Figure 2).

In February 2020, the patient referred to the gastroenterology clinic. Colonoscopy performed on February 27, 2020, found a 2 x 2 cm polypoid lesion in the ascending colon, proximal to the hepatic flexure. The lesion was ulcerated, no bleeding was present, and biopsies were obtained by cold forceps for histology.

The patient referred to the general surgery clinic in March 2020. The patient was asymptomatic. On physical examination, the abdomen was soft and lax, with no tenderness, past surgical history of ovarian cystectomy, no visible scars, or hernias.

Routine laboratory tests revealed normocytic hypochromic anemia, low hemoglobin (10.3 gram/dl: normal 13-17 gram/dl), low red blood cells (3.78 M/UL: normal 4.8-6.4 M/UL), coagulation profile; prothrombin (PT) (13.0 seconds; normal, 9.4-12.5 seconds), low platelet count (141×109/L: normal 150 to 400 × 109/L), and low white blood cells. However, the cause of pancytopenia are most likely due to the side effects of chemotherapy. Liver function tests, urea, and creatinine within the normal range. The carcinoembryonic antigen (CEA) was (2.06 ng/ml: normal range 0-5 ng/ml).

After reviewing the radiological investigations, the tumor, nodes, metastasis (TNM) classification was cT1N0M0. The discussion with colorectal surgeons and the decision necessitated laparoscopic right hemicolectomy. Due to the small tumor size, preoperative colonoscopy was performed to localize the tumor site with India Ink, which was done successfully on April 2, 2020. Due to the COVID-19 pandemic, there were difficulties in admitting the patient and preparing for the surgical procedure even when the COVID-19 swab came negative. Fortunately, the patient was admitted on May 12, 2020, to the surgical ward for elective right hemicolectomy.

Laparoscopic right hemicolectomy with a colonic mesocolic excision was performed. The India Ink site identified the mid-ascending colon, and the tumor was recognized by the hand of the surgeon. A right hemicolectomy was performed with side-to-side extracorporeal ileocolonic anastomosis.

**Figure 3:**
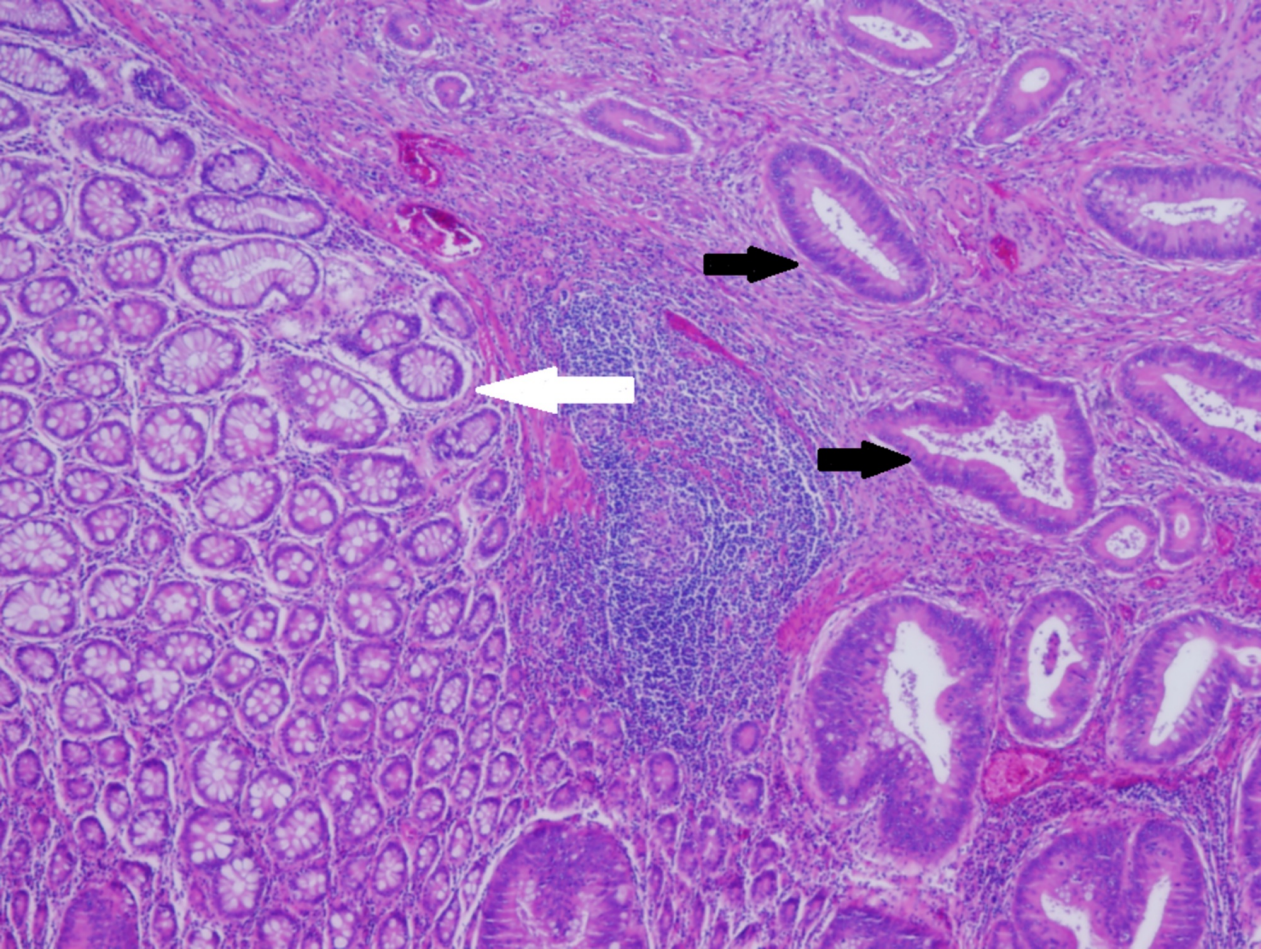
Histopathological examination revealed healthy cells (white arrow) alternating with malignant adenocarcinoma (black arrow) invading the muscularis propria

In the first postoperative days, the patient was vitally stable despite the gradually dropping hemoglobin (7.3 g/dl; normal 13-17 g/dl); she had bleeding from the anastomotic site and passed a dark stool, which presented as melena. She was treated conservatively with a one-unit pack of red blood cells. The following day, the hemoglobin raised to 8.5 g/dl. The patient was discharged four days after surgery with a full diet and regular bowel motion. Histopathological examination of the right colon lesion showed low-grade adenocarcinoma, 3.0 cm * 0.6 cm in size invading the muscularis propria; 30 lymph nodes were examined and no lymphovascular invasion was detected (Figure 3).

The two-week and then one-month postoperative followup with general surgery was uneventful, and the patient was in her usual state of health. Oncology follow-up advised computed tomography (CT) chest, abdomen, and pelvis and checking tumor marker at six months post-operation.

## Discussion

Colon cancer is one of the major health concerns worldwide, with rising incidence and death rates in developing areas [6]. Approximately one million cases are diagnosed annually, representing 10% of cancer diagnosed annually [7]. About 694,000 deaths from colon cancer occur every year. In Saudi Arabia, colon cancer is the highest form of malignant tumor among Saudi men and the third among Saudi women according to the Saudi Cancer Registry Incidence Report [8].

After reviewing the Surveillance, Epidemiology, and End Results (SEER) and Cancer Statistics Review (CSR) from 1975-2014, the average age at diagnosis of colon cancer worldwide is 68 for men and 72 for women [9]. The majority of colon cancer cases present as sporadic (up to 70%) while a few cases present as familial or inherited (about 30%). CRC has a diversity of risk factors, including food intake (e.g., low fiber, highly processed/red meat, and high caloric value), obesity, low physical activity, smoking, socioeconomic and educational state, and exposure to radiation [10]. The nonmodifiable risk factors of colon cancer include a history of colon cancer in the patient's family, age at the time of diagnosis of colon cancer, and race or ethnicity of the patient. There is no clear presentation of colon cancer. However, a change in bowel habits, losing weight, and anemia could guide toward colon cancer before it complicates to a total intestinal obstruction or perforation with progression and metastasis of advanced tumors [1]. One out of four patients with colon cancer present with metastasis at the time of diagnosis [5]. The gold standard for the diagnosis of a colon lesion is a full colonoscopy reaching the cecum with a biopsy taken for histopathological confirmation [5].

FDG/PET can detect high abnormality uptake throughout the body organs; a high uptake requires intensive work-up. A retrospective study was conducted on 3000 patients, known cases of cancer or at a high risk getting it; about 7% were found to have incidental cancers, commonly affecting the colon, lung, and stomach. False-positive results were acceptable, reaching 4.5% for all patients. Inflammatory markers, such as neutrophils, lymphocytes, and macrophages, increase FDG uptake, leading to highly false-positive results in patients with infection or inflammation in that organ [11-12]. In Pakistan, a study was conducted to detect the primary site of cancer of patients diagnosed with carcinoma of unknown primary (CUP). It showed a detection rate of 57%, with 68% sensitivity and a 76% positive predictive value [13]. In workup for patients with advanced breast cancer to detect metastasis, FDG/PET was superior over the CT scan to detect other axillary lymph node metastasis. On the other hand, FDG/PET is inferior to bone scintigraphy to detect bone metastasis [14]. As advised by the ENT team in our hospital and by Abouzied et al. [15], FDG/PET is better than any other imaging modality for the staging and follow-up of head and neck cancer.

No evidence supports the routine clinical use of FDG/PET colonography in diagnosing colorectal cancer for many reasons, most importantly, cost and availability [16]. However, evidence showed that FDG/PET has higher sensitivity, specificity, and accuracy than contrast-enhanced computed tomography (CECT) in detecting recurrent or metastatic disease [17]. Furthermore, this nuclear medicine imaging has a role in monitoring therapy by detecting the tumor's metabolic and morphological changes in the early stages [18], regardless of FDG/PET limitation and the small size of the mass that needed localizing preoperatively by India Ink. The timing of the whole management in the era of COVID-19 was a bit difficult but, most importantly, successful.

## Conclusions

During the COVID-19 circumstances, the patient was clinically stable when we discovered her incidental colon finding. In addition, her condition was not emergent. FDG/PET is valuable nuclear imaging in oncological settings. Early detection of abnormal uptake requires intensive work-up; thus, the patients' survival and prognosis are relatively long. The colon, as well as rectum and lung, are favorable for the uptake of FDG, but to some organs like a bone, it may be challenging. In our case, incidental findings of uptake unrelated to primary cancer workup are uncommon. However, prompt management of this finding is crucial for the patient’s healthy life expectancy.
